# Antimicrobial efficacy against *Pseudomonas aeruginosa* biofilm formation in a three-dimensional lung epithelial model and the influence of fetal bovine serum

**DOI:** 10.1038/srep43321

**Published:** 2017-03-03

**Authors:** Aurélie Crabbé, Yulong Liu, Nele Matthijs, Petra Rigole, César De La Fuente-Nùñez, Richard Davis, Maria A. Ledesma, Shameema Sarker, Rob Van Houdt, Robert E. W. Hancock, Tom Coenye, Cheryl A. Nickerson

**Affiliations:** 1 Laboratory of Pharmaceutical Microbiology (LPM), Ghent University, Ghent, Belgium; 2 The Biodesign Institute, Center for Infectious Diseases and Vaccinology, Arizona State University, Tempe, Arizona, United States of America; 3 University of British Columbia, Centre for Microbial Diseases and Immunity Research, Vancouver, British Columbia, Canada; 4 Unit of Microbiology, Belgian Nuclear Research Centre (SCK·CEN), Mol, Belgium; 5 School of Life Sciences, Arizona State University, Tempe, Arizona 85287, United States of America

## Abstract

*In vitro* models that mimic *in vivo* host-pathogen interactions are needed to evaluate candidate drugs that inhibit bacterial virulence traits. We established a new approach to study *Pseudomonas aeruginosa* biofilm susceptibility on biotic surfaces, using a three-dimensional (3-D) lung epithelial cell model. *P. aeruginosa* formed antibiotic resistant biofilms on 3-D cells without affecting cell viability. The biofilm-inhibitory activity of antibiotics and/or the anti-biofilm peptide DJK-5 were evaluated on 3-D cells compared to a plastic surface, in medium with and without fetal bovine serum (FBS). In both media, aminoglycosides were more efficacious in the 3-D cell model. In serum-free medium, most antibiotics (except polymyxins) showed enhanced efficacy when 3-D cells were present. In medium with FBS, colistin was less efficacious in the 3-D cell model. DJK-5 exerted potent inhibition of *P. aeruginosa* association with both substrates, only in serum-free medium. DJK-5 showed stronger inhibitory activity against *P. aeruginosa* associated with plastic compared to 3-D cells. The combined addition of tobramycin and DJK-5 exhibited more potent ability to inhibit *P. aeruginosa* association with both substrates. In conclusion, lung epithelial cells influence the efficacy of most antimicrobials against *P. aeruginosa* biofilm formation, which in turn depends on the presence or absence of FBS.

The opportunistic pathogen *Pseudomonas aeruginosa* is one of the leading causes of acute and chronic respiratory infections^1^,^2^,^3^,^4^,^5^. Intrinsic, acquired, and adaptive antibiotic resistance makes *P. aeruginosa* particularly challenging in the clinic^6^. Furthermore, during chronic lung infections, such as in individuals with cystic fibrosis, *P. aeruginosa* forms biofilms – a phenotype that contributes to high (adaptive) antibiotic resistance and mediates long-term host colonization^7^,^8^. Therefore, novel antimicrobial approaches that target bacterial virulence factors (such as those regulating biofilm formation) or host factors (such as immune stimulation) represent promising alternatives to antibiotics, especially given the global problem of antibiotic resistance^9^,^10^. When assessing the clinical potential of novel antimicrobial agents, mimicking the *in vivo* bacterial phenotype and incorporating key aspects of host tissues are essential^11^,^12^,^13^. Indeed, the more closely *in vivo* host-pathogen interactions are reflected in a model system, the more relevant the experimental outcome will be to the patient. During the natural course of infection, mucosal pathogens encounter host factors that can act as regulatory signals, and may be beneficial or detrimental for the initiation and development of a successful infection^11^,^12^,^14^,^15^,^16^,^17^,^18^. The collection of host biochemical cues and biophysical forces that pathogens might encounter during an infection is termed the host microenvironment. The microenvironment includes multiple host cell types, factors produced by these cells (such as cytokines from epithelial cells and other innate immune cells) or resident microbiota, extracellular matrix (ECM) components (proteins, growth factors, and other signaling molecules), and physical forces (such as tensile forces and fluid-shear). While it is well known that certain host factors (such as stress hormones) affect the growth, biofilm formation and virulence of *P. aeruginosa*^19^,^20^, there is still limited knowledge on the influence of lung epithelial cells on the efficacy of antimicrobial agents. An additional factor to consider when studying the influence of host cells on antimicrobial agent efficacy *in vitro* is the cell culture medium. While cell culture media have been developed to mimic the chemical composition of the *in vivo* microenvironment, they contain components that are not necessarily found at the mucosal barrier but may be important for cell growth and differentiation. One such compound is fetal bovine serum (FBS), which is a conventional cell culture supplement needed for the growth of many cell lines, including for lung adenocarcinoma A549 cells^21^,^22^. This is of particular interest when evaluating antimicrobial agent efficacy as serum has been shown to inhibit the efficacy of antibiotics and peptides^23^,^24^.

Three-dimensional (3-D) lung epithelial models have been shown to reflect key aspects of the parental tissue, including 3-D architecture, barrier function, apical-basolateral polarity, secretion of mucins, and multicellular complexity^11^,^21^,^22^,^25^. All of these phenotypic traits are of importance in mucosal immunity, and play a role during interactions of *P. aeruginosa* with the host^12^. An established approach to generate organotypic 3-D lung models is using a low fluid-shear optimized suspension culture technology, designated as the rotating wall vessel (RWV) bioreactor. Previously, a RWV-derived 3-D lung epithelial model was developed by culturing A549 cells on the surface of collagen I-coated porous microcarrier beads^21^. In addition to the above-mentioned *in vivo*-like characteristics of normal cells, the 3-D epithelial model that was derived from A549 cells showed lower expression of cancer markers when compared to conventional A549 monolayers, thus reflecting a more normal differentiated tissue phenotype. Using the 3-D A549 lung model, Carterson *et al*. (2005) demonstrated an infection profile with *P. aeruginosa* that was more relevant to the *in vivo* scenario, with regards to bacterial adhesion and invasion, and host-secreted cytokines. Three-dimensional cell culture models of other tissues that mimic *in vivo*-like characteristics have been found to mimic drug sensitivity patterns observed *in vivo*, including penetration into target tissues and drug resistance, and to more closely parallel patient responses to treatment as compared to conventional monolayers and *in vivo* animal models^26^,^27^,^28^,^29^,^30^. However, to our knowledge, RWV-derived 3-D cell culture models have not yet been applied for testing the efficacy of antimicrobial agents in the context of infectious diseases.

This study applies the 3-D A549 lung epithelial model as a novel platform to study the influence of lung epithelial cells on antimicrobial agent efficacy against a clinically problematic microorganism. The objectives of this study were three-fold: (i) Development of an approach to obtain *P. aeruginosa* biofilms associated with 3-D A549 lung epithelium, to more closely approximate both the *in vivo* bacterial and host phenotypes, (ii) Examination of the influence of lung epithelial cells on the ability of commonly used antibiotics as well as a novel antimicrobial agent to inhibit the initial stage of biofilm formation by comparing adhesion/association with 3-D epithelial cells and with a plastic surface, and (iii) Assessment of the influence of a conventional cell culture supplement (FBS) on the obtained results. The novel antimicrobial agent tested was the anti-biofilm peptide DJK-5, which inhibits biofilm formation and eradicates preformed biofilms constructed from a wide range of microorganisms, including *P. aeruginosa*, on abiotic surfaces and invertebrate *in vivo* models^31^. DJK-5 is a D-enantiomeric cationic peptide that exerts its anti-biofilm function through degradation of the stringent stress response signal ppGpp^31^. Synergistic activity between DJK-5 and several antibiotics (including tobramycin) has been demonstrated^31^.

In the present study, we found that *P. aeruginosa* formed biofilms on 3-D A549 lung epithelial cells that could not be eradicated with high concentrations of antibiotics, hereby mimicking aspects of the *in vivo* bacterial phenotype. Biofilms associated with 3-D lung epithelial cells did not affect epithelial cell viability for at least seventeen hours, or the formation of tight junctions for at least six hours of infection. Then, our novel biofilm approach was used to study *P. aeruginosa* biofilm inhibition by antimicrobial agents in a model system that mimics aspects of the *in vivo* host tissue, which may be of importance when investigating the early stages of host-pathogen interactions in the context of establishing chronic lung disease (such as in patients with cystic fibrosis). Experiments were conducted both in the presence and absence of 10% FBS to evaluate the effect of this commonly used cell culture supplement on differential antimicrobial agent efficacy. The results point to significant differences between the efficacy of certain antibiotics and DJK-5 against *P. aeruginosa* biofilms formed on 3-D lung epithelial cells as compared to a plastic surface. In addition, we report that the differential efficacy of certain antibiotics and DJK-5 was influenced by the presence of FBS. These findings highlight the role of the host and its microenvironment in the efficacy of antimicrobial agents, as well as the usefulness of the 3-D lung biofilm model as a complementary tool for the evaluation of novel antimicrobial compounds.

## Results

### *P. aeruginosa* formed biofilms on 3-D lung epithelium

**Figure 1:**
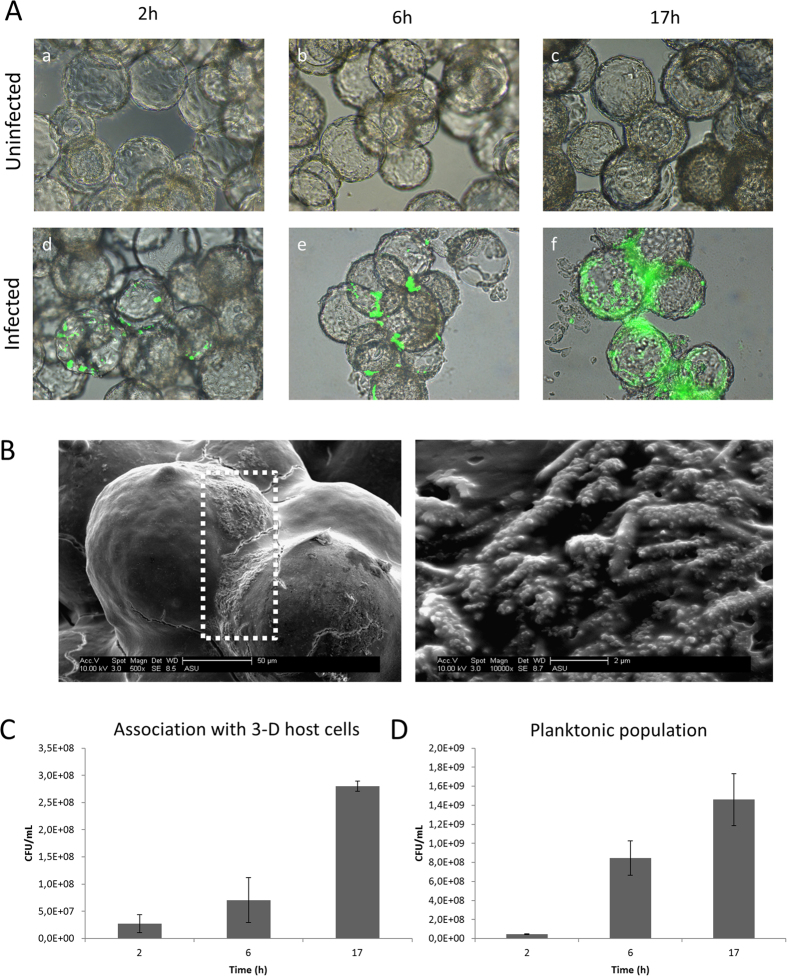
*P. aeruginosa* formed biofilm-like structures on 3-D lung epithelial cells. **(A)** 3-D lung epithelial cells co-cultured without (a–c) or with (d–f) GFP-expressing *P. aeruginosa* PAO1 for 2 h, 6 h and/or 17 h. Immunofluorescence and light microscopy images at a magnification of 400x are overlaid. **(B)** Scanning electron microscopy images of *P. aeruginosa* biofilms (white dotted frame) at the 6 h infection time point with a magnification of 500x (left image) and 10,000x (right image) (**C)** Number of colony forming units (CFUs) adhering to the 3-D lung epithelial cells at different time points. **(D)** Number of CFUs present in the surrounding medium (not associated to host cells).

GFP-expressing *P. aeruginosa* microcolonies were observed at the apical surface of 3-D A549 epithelial cells after 2 h and 6 h of infection (Fig. 1A, panels d and e). At 17 h post-infection, biofilm-like structures covered a large part of the 3-D epithelial cell surface (Fig. 1A, panel f). The formation of biofilm-like structures was confirmed by scanning electron microscopy (SEM) analysis at the 6 h time point, and indicated the association of bacteria in a matrix (Fig. 1B). In addition, the number of host-associated *P. aeruginosa* cells increased over time, and the increase in host-associated bacterial numbers was different from the increase in the number of planktonic cells between 2 h and 6 h of infection (Fig. 1C,D). Indeed, the host-associated *P. aeruginosa* population increased on average by 2.6-fold between 2 h and 6 h, while the bacterial population that was not host-associated increased 18.0-fold between 2 h and 6 h (p < 0.05). Between 6 h and 17 h of infection, the increase in the host-associated bacterial population and planktonic populations was not significantly different (p > 0.05). This suggests that the increase in host-associated *P. aeruginosa* may in part be independent of growth of the planktonic bacteria present in the surrounding cell culture medium.

### *P. aeruginosa* biofilms on 3-D lung epithelium did not affect host cell viability for up to 17 h of infection

**Figure 2:**
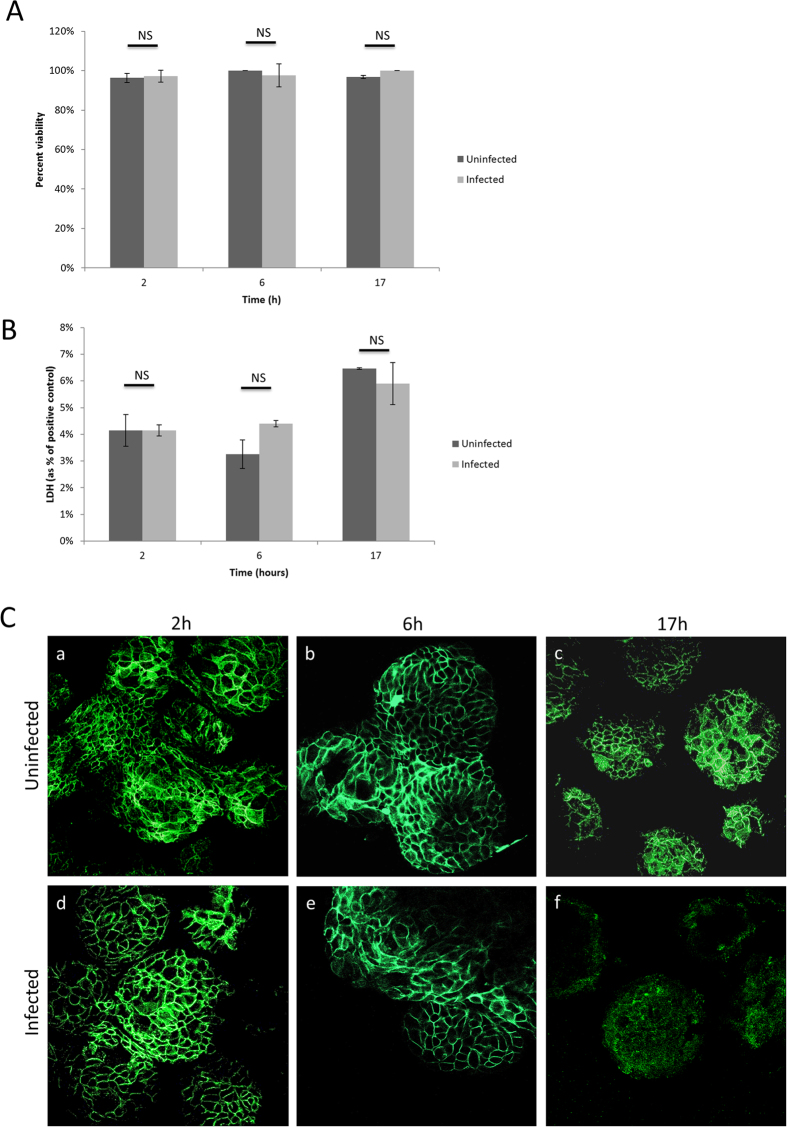
Effect of *P. aeruginosa* biofilms on host cell viability and tight junction formation in 3-D lung epithelial cells, following infection for 2 h, 6 h, and 17 h (as compared to uninfected controls): (**A**) Trypan blue exclusion assay. (**B**) LDH release assay. LDH release is presented as a percentage of a positive control (3-D lung epithelial cells lysed with Triton-X100). (**C**) Immunofluorescence imaging of the tight junctional marker β-catenin (green) for uninfected controls (a–c) and infected samples (d–f), at a magnification of 400x. NS = non-significant (p > 0.05).

We observed that *P. aeruginosa* biofilms associated with 3-D lung epithelial cells did not significantly affect host cell viability for up to 17 h post-infection, as determined by assessing Trypan blue exclusion (more than 97% viability) (Fig. 2A) and cytosolic lactate dehydrogenase (LDH) release (less than 7% cell death) (Fig. 2B).

### Tight junctions were maintained during initial infection time points

The overall integrity of the 3-D tissue was maintained over the 17 h duration of the experiment, with limited detachment of the cells from their micro-carrier bead scaffolds (Fig. 1A, panels a-c for uninfected cells, panels d-f for infected cells). Starting at 18 h post-infection, cells significantly detached from the scaffold (Supplementary Figure 1). We assessed whether epithelial barrier function was maintained during the infection process by immunofluorescence profiling of the tight junctional protein β-catenin. A honey-comb expression pattern of β-catenin was observed at 2 h and 6 h post-infection, comparable to the matched time point non-infected control (Fig. 2C). At the 17 h infection time point, the pattern of expression of β-catenin was lost (Fig. 2C), which might explain the subsequent detachment of host cells from their scaffold at later time points. Accordingly, subsequent biofilm studies were performed at 6 hours.

### Biofilms formed on 3-D lung epithelial cells and on plastic could not be eradicated with high concentrations of antibiotics

**Figure 3:**
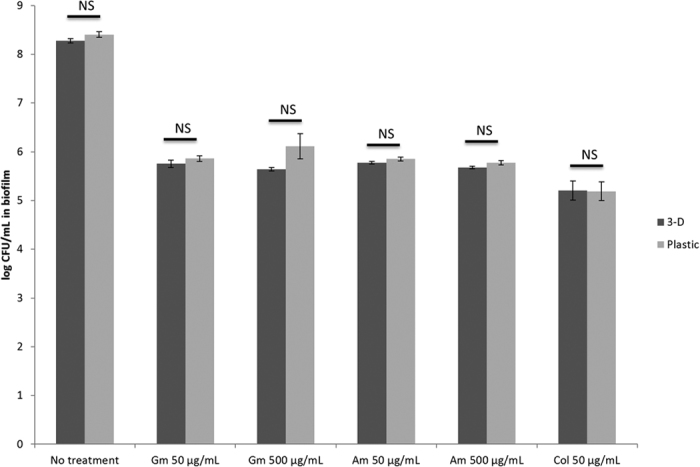
*P. aeruginosa* biofilms associated to 3-D lung epithelial cells could not be eradicated with high concentrations of antibiotics. *P. aeruginosa* biofilms formed after 6 h on plastic or 3-D lung epithelial cells were exposed to high concentrations of gentamicin (50 μg/mL = 3x MIC, 500 μg/mL = 31x MIC), amikacin (50 μg/mL = 6x MIC, 500 μg/mL = 60x MIC) and colistin (50 μg/mL = 12.5x MIC) for an additional 17–18 h to assess their susceptibility to antibiotics. Control biofilms on plastic and 3-D cells were incubated for the same duration without antibiotics. Gm = Gentamicin, Col = colistin, Am = amikacin. NS = non-significant (p > 0.05).

Next, we tested the susceptibility of biofilms associated with 3-D lung cells and with plastic to antibiotics. For this purpose, biofilms grown for 6 h on 3-D lung epithelial cells or on plastic were exposed to high concentrations of antibiotics (gentamicin, amikacin, and colistin; between 3-fold and 60-fold the MIC). Neither biofilms associated with 3-D lung epithelial cells nor those associated with plastic could be eradicated using high concentrations of the tested antibiotics (Fig. 3) [for gentamicin at 50 μg/mL (3x MIC) and 500 μg/mL (31x MIC), for amikacin at 50 μg/mL (6x MIC) and 500 μg/mL (60x MIC), and for colistin at 50 μg/mL (12.5x MIC)].

### The biofilm-inhibitory activity of conventional antibiotics against *P. aeruginosa* was altered when biofilms were formed on 3-D lung epithelial cells compared to plastic

**Figure 4:**
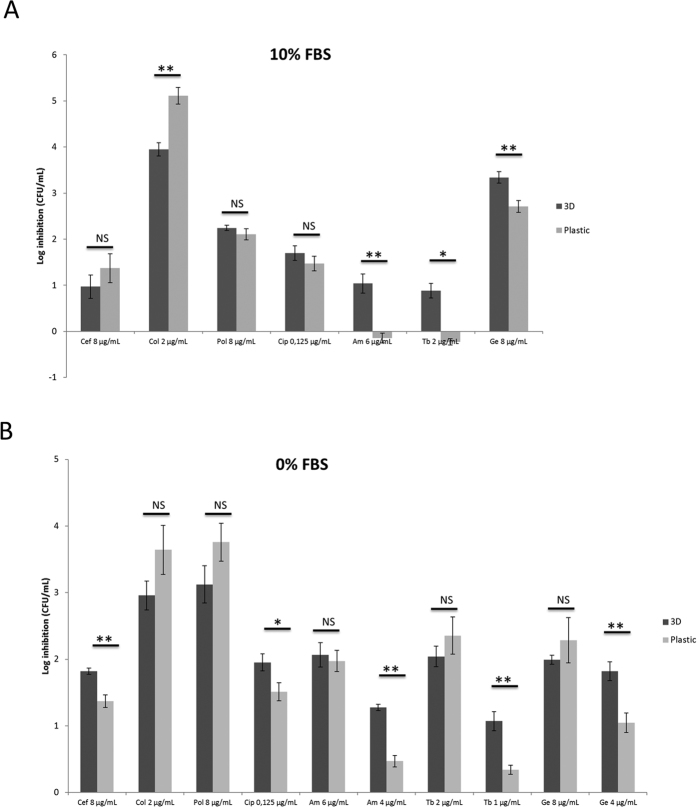
Inhibition of *P. aeruginosa* association with 3-D lung epithelial cells or plastic by conventional antibiotics. Inhibition of association by the antimicrobial compounds was tested in cell culture medium that contained 10% FBS (**A**) or in medium without FBS (**B**). Data is presented as log inhibition of bacterial association compared to the control that received no treatment. Cef = ceftazidime, Col = colistin, Pol = polymyxin B, Cip = ciprofloxacin, Am = amikacin, Tb = tobramycin, Ge = Gentamicin. *p < 0.05, **p < 0.01, NS = non-significant (p > 0.05). Standard error bars are presented, n ≥ 3.

We applied the 3-D lung biofilm model to assess the role of differentiated host cells on the efficacy of antibiotics that are commonly used to treat chronic *P. aeruginosa* lung infections. For these experiments, cell culture medium was used that contained 10% FBS (the same as that used for the generation of the 3-D lung epithelial model). The biofilm-inhibitory activity of antibiotics commonly used to treat cystic fibrosis patients was determined based on the number of bacteria (CFU/mL) that associated with 3-D lung epithelial cells or plastic in the presence or absence of antibiotics over a period of six hours. All three aminoglycosides tested (i.e., tobramycin, amikacin and gentamicin) more effectively inhibited *P. aeruginosa* association with 3-D cells than with plastic (Fig. 4A). In contrast, colistin less effectively inhibited *P. aeruginosa* association with 3-D lung cells compared to plastic. The effects of another polymyxin (polymyxin B), ceftazidime and ciprofloxacin were similarly effective in both test conditions (Fig. 4A).

### FBS influences the differential efficacy of antimicrobial agents against biofilms on 3-D lung epithelial cells or plastic

Next, the influence of FBS was assessed on the differential efficacy of antibiotics against biofilms associated with 3-D lung epithelial cells or plastic. First, select key phenotypic characteristics of the new biofilm approach were validated in medium without FBS for the 6 h incubation time point. The data demonstrates that FBS does not strongly influence (i) the bacterial association (log CFU/mL) to 3-D lung epithelial cells or plastic in the absence of antimicrobial agents, (ii) LDH release following infection, (iii) formation of biofilm-like structures on both models, and (iv) reduced susceptibility of the biofilms to most tested antibiotics (Supplementary data Fig. 2). For the latter, the only exception is colistin, to which biofilms associated with 3-D lung epithelial cells and plastic were more susceptible in the absence of FBS (~3 logs survival) compared to that observed in the presence of FBS (~5 logs survival), However, the high resistance to the other antibiotics suggests that bacteria grown in medium without FBS also form biofilms. It was observed that FBS influences the efficacy of certain antibiotics against biofilm formation on plastic and/or 3-D cells (Supplementary Figure 3).

With regard to the differential efficacy of antibiotics against biofilms associated with 3-D lung epithelial cells or plastic, most of the tested antibiotics showed an enhanced efficacy in the presence of 3-D lung epithelial cells in FBS-free medium (Fig. 4B), with the exception of the polymyxins (colistin and polymyxin B) for which no differences between either of the two models were observed. Of note, we initially tested the same antibiotic concentrations as those used for medium with FBS (i.e., concentrations were chosen to obtain at least one log of inhibition for biofilms associating with 3-D cells). For the aminoglycosides, the additive effect of 3-D epithelial cells could not be observed at these concentrations. However, when lowering the aminoglycoside concentration, 3-D lung epithelial cells enhanced the efficacy of these antibiotics (Fig. 4B), as was observed in medium with FBS (Fig. 4A).

### The anti-biofilm peptide DJK-5 had a differential efficacy in inhibiting *P. aeruginosa* association with 3-D lung epithelial cells and plastic, and its efficacy was influenced by fetal bovine serum

**Figure 5:**
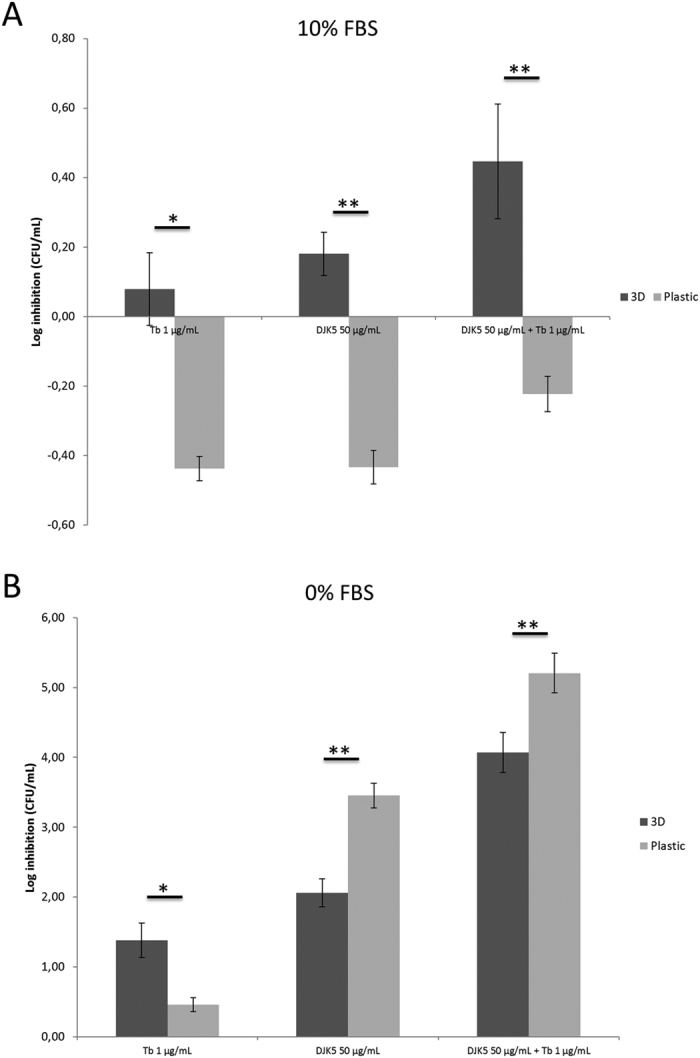
Inhibition of *P. aeruginosa* association with 3-D lung epithelial cells versus plastic by the anti-biofilm peptide DJK-5, tobramycin and their combination. Inhibition of association of bacteria with these surfaces by the antimicrobial compounds was tested in cell culture medium that contained 10% FBS (**A**) or no FBS (**B**). Data is presented as log inhibition of bacterial association compared to the control that received no treatment. Tb = tobramycin. *p < 0.05, **p < 0.01. Standard error bars are presented, n ≥ 3.

The efficacy of the recently developed anti-biofilm peptide DJK-5 was tested, alone and in combination with the antibiotic tobramycin. The efficacy of DJK-5, tobramycin and their combination was initially assessed, after 6 hours, in influencing *P. aeruginosa* association with 3-D lung epithelial cells and plastic in cell culture medium (GTSF-2) containing 10% FBS. Bacterial association with 3-D cells or plastic was slightly affected by 50 μg/mL DJK-5 (Fig. 5A), a concentration that corresponds to ~50x the MBIC (Minimal biofilm inhibitory concentration, tested on plastic) in BM2 minimal medium^31^. Despite the slight effect of DJK-5 in the presence of 10% FBS, this antimicrobial agent significantly decreased the bacterial association with 3-D lung epithelial cells (p < 0.05) when compared to the no treatment control, both with (2.8-fold, 64% decrease) and without tobramycin (1.5-fold, 33% decrease). At the tested concentration, tobramycin alone did not significantly inhibit the formation of *P. aeruginosa* biofilms associated with 3-D cells compared to the no treatment control (p > 0.05). The effect of tobramycin and DJK-5 on 3-D cells appeared to be additive (Fig. 5A). In contrast, a modest (<2-fold) increase in association with a plastic surface was observed with all agents including their combination (Fig. 5A).

Since serum is not present in the natural environment of epithelial cells and has been shown to affect the efficacy of certain antimicrobial agents, including peptides^23^, the experiment was repeated in cell culture medium that did not contain FBS (0% FBS). Of note, the concentration of tobramycin was slightly adjusted to obtain similar biofilm-inhibitory effects in the 0% (1 μg/mL) (Fig. 5B) and 10% (2 μg/mL) (Fig. 3) medium conditions.

In the absence of FBS, DJK-5 exerted potent inhibition of *P. aeruginosa* association with 3-D lung epithelial cells and with plastic, reducing cell numbers by 100- and 3,000-fold respectively (Fig. 5B). Stronger inhibitory activity of DJK-5 against *P. aeruginosa* association with plastic was observed as compared to 3-D lung epithelial cells, both in the absence and presence of tobramycin (Fig. 5B). The combined activity of tobramycin and DJK-5 was higher (resulting in a 10,000 to 150,000-fold reduction in bacterial counts associated with plastic and 3-D cells, respectively) than the sum of the activity of the individual agents, both for association with 3-D cells and plastic (p < 0.05 and p < 0.01, respectively).

### DJK-5 demonstrated biofilm-inhibitory activity on plastic and 3-D cells

As shown in Fig. 5, DJK-5 inhibited *P. aeruginosa* association with 3-D lung epithelial cells after 6 hours, both in the presence and absence of 10% FBS. We subsequently determined whether DJK-5 inhibited biofilm formation at 6 hours on 3-D lung epithelial cells and plastic using microscopy for the qualitative assessment of biofilm formation, based on the observation of fluorescent cell clusters.

**Figure 6:**
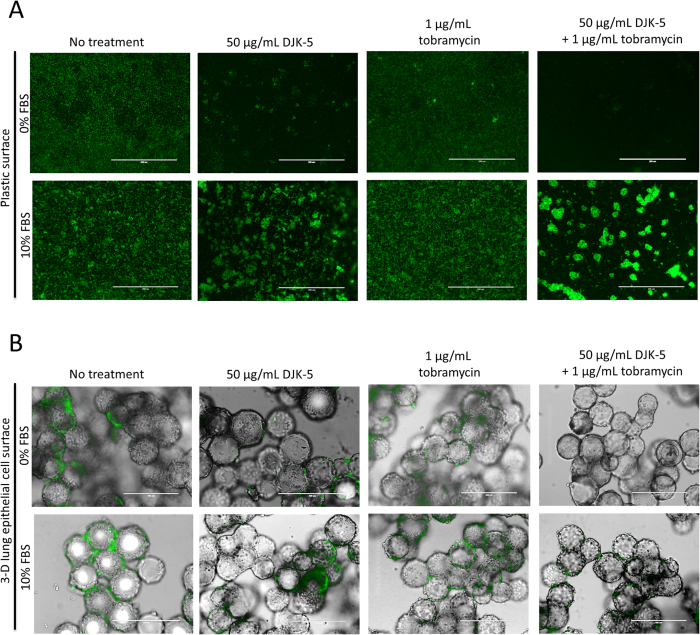
Qualitative assessment of antimicrobial agent activity against *P. aeruginosa* biofilm formation on plastic (**A**) or 3-D lung epithelial cells (**B**), in the presence and absence of 10% FBS. Epifluorescence (**A**) and an overlay of light microscopy and epifluorescence images (**B**) were obtained at a magnification of 300x. Scale bar = 400 μm.

In no FBS medium, DJK-5 alone and in combination with tobramycin showed strong inhibitory activity in blocking the formation (or increasing the dispersal) of biofilms on plastic (Fig. 6A, upper panels) and 3-D cells (Fig. 6B, upper panels). Even in the presence of 10% FBS, the combination of DJK-5 and tobramycin substantially reduced the formation of *P. aeruginosa* biofilms on 3-D lung epithelial cells (Fig. 6B, lower panels). Biofilms formed on plastic in the presence of DJK-5 and tobramycin in 10% FBS medium were drastically altered in structure, with compact patchy cell clusters as opposed to a more homogenous surface coverage in the untreated control (Fig. 6A). These findings suggested that despite differences in the efficacy of DJK-5 in inhibiting bacterial association with plastic and 3-D cells (Fig. 5), biofilm-inhibitory activity occurred under both circumstances (Fig. 6).

## Discussion

Accurately predicting the efficacy of antimicrobial agents for treatment of bacterial infections will be improved by the availability of model systems that replicate both the bacterial and host *in vivo* phenotypes^11^,^12^,^13^. In the present study, we developed a novel approach to study interactions between *P. aeruginosa* biofilms and biotic surfaces, by generating biofilms associated with *in vivo*-like 3-D lung epithelial cells. The 3-D lung epithelial model was previously shown to have phenotypic traits of the parental tissue, including barrier function, polarity, and multicellular complexity^21^,^22^,^25^. Not only are these phenotypic characteristics essential for mucosal immunity, they also play an important role in the host colonization of *P. aeruginosa*^12^,^32^,^33^,^34^. For example, polarized epithelial cells are relatively resistant to *P. aeruginosa* invasion, in contrast to non-polarized, damaged epithelial cells in which invasion typically can be observed^33^,^35^,^36^,^37^. Therefore, it is anticipated that *in vitro* model systems that mimic aspects of the parental tissue will result in a colonization profile of *P. aeruginosa* that better reflects the *in vivo* infectious disease scenario. Indeed, previous work demonstrated that a 3-D colonic epithelial model infected with the enteric pathogen *Salmonella enterica* serovar Typhimurium replicated the *in vivo* disease-associated characteristics of both the host and the pathogen^38^,^39^.

Besides mimicking the phenotypic traits of lung tissue, our approach also replicates the biofilm phenotype of *P. aeruginosa* that is typically observed during chronic lung infections^7^. *P. aeruginosa* formed biofilm-like clusters on the 3-D lung epithelial cells that increased in size over the duration of the infection. The biofilm-like structures could not be eradicated with high concentrations of antibiotics, a well-described feature of biofilms^7^, which further indicates that the observed structures were indeed biofilms. Biofilm cells were encased in a matrix, which could be derived from the host and/or the bacteria. The 3-D lung epithelial model used in this study has a well-defined apical mucus layer that contains the respiratory mucins MUC5AC and MUC1^21^,^22^. Since host-produced factors, such as mucins, play a role in *P. aeruginosa* biofilm formation^40^, the presence of a mucus layer in the 3-D lung epithelial model may have contributed to biofilm formation by *P. aeruginosa*. It remains to be determined whether *P. aeruginosa* biofilms are attached to the epithelial cells or are present as non-attached aggregates in the mucus layer of the lung epithelial cells; the latter is a phenotype observed in the lung mucus of cystic fibrosis patients^41^. Of note, both biofilm phenotypes have previously been demonstrated to exert similar responses to antibiotics and phagocytes^42^.

A key hallmark of biofilms is their long-term presence on mucosal surfaces. A major challenge with studying *P. aeruginosa* biofilms *in vitro* in the presence of host cells is massive host cell death and detachment from the scaffold following 6–8 hours of infection, which precludes longer term biofilm studies^43^,^44^,^45^. These previous studies generated *P. aeruginosa* biofilms on host cells grown on plastic or on filter inserts. In contrast, generating *P. aeruginosa* biofilms on 3-D lung epithelial cells did not result in cell death or significant cell detachment from the microcarrier bead scaffolds over a period of seventeen hours. However, at the 17 h time point, barrier function was lost, as determined by imaging of the tight junctional marker β-catenin. Indeed, *P. aeruginosa* produces a range of virulence factors that damage barrier function^46^,^47^,^48^. In agreement with our findings, 3-D cell cultures of different cell lines demonstrated enhanced robustness and/or recovery following infection, as compared to their respective monolayer controls^21^,^38^,^49^.

Use of the 3-D lung epithelial model for studying antibiotic efficacy against *P. aeruginosa* biofilm formation revealed significant differences with a static abiotic biofilm model, which is one of the most commonly used biofilm assays. It is important to note that current MIC assays that are often used for drug discovery do not mimic the host environment and thus have somewhat limited predictive value^50^. While we consider that the system described here is closer to the *in vivo* situation, it is currently not suitable as a primary screening system but might have value as a secondary screen.

Previous studies demonstrated that established biofilms associated with different types of epithelial cells consistently demonstrated high resistance to antibiotics^43^,^44^,^45^,^51^, a phenotype that we confirmed in the present study. In addition, *P. aeruginosa* biofilms pre-formed on cystic fibrosis bronchial epithelial (CFBE) cells were previously found to exert enhanced tolerance to antimicrobial agents as compared to biofilms grown on plastic^43^,^44^. A recent study demonstrated that the synergistic effects of mannitol in combination with tobramycin for eradication of *P. aeruginosa* biofilms pre-formed on a plastic surface could not be observed on biofilms pre-formed on CFBE cells^52^. Recently, biofilm formation by *P. aeruginosa* was found to be inhibited by an antimicrobial peptide (WLBU2) both when biofilms were formed on plastic or on CFBE cells^53^. However, this study did not compare the peptide efficacy in both model systems.

We found that when *P. aeruginosa* associated with host cells as compared to a plastic surface, the efficacy of certain antimicrobial agents was different at inhibiting the formation of biofilms. Moreover, the differential efficacy on plastic versus host cells of certain antibiotics depended on the presence or absence of FBS (a conventional cell culture supplement) in the culture medium. With the exception of the polymyxins, the tested antibiotics showed enhanced efficacy in the presence of 3-D lung epithelial cells, in medium with FBS, in medium without FBS, or in both media. It could be hypothesized that antimicrobial compounds produced by respiratory epithelial cells (including A549 cells)^54^,^55^, such as defensins, may act in synergy with these antibiotics^56^,^57^. However, the expression of antimicrobial compounds in the 3-D A549 lung model will need to be determined to assess their potential role in the observed increased efficacy of aminoglycosides.

FBS inhibited the efficacy of DJK-5 and several antibiotics at the initial bacterial association level. In agreement with these findings, serum was previously found to inhibit the activity of antibiotics and cationic peptides^23^,^24^. Apart from the relevance of these findings in the choice of cell culture media and supplements when studying antimicrobial agent efficacy *in vitro*, they might also be relevant for cystic fibrosis patients with hemoptysis (~4%)^58^. Indeed, the presence of blood in the respiratory tract of these patients may influence the efficacy of antimicrobial agents. Nevertheless, the inhibition of bacterial association with epithelial cells by DJK-5 could still be observed in the presence of 10% FBS, and there appeared to be an additive effect with tobramycin. This might be related to the strong ability of DJK-5 to promote biofilm dispersal^31^ since dispersed cells, being planktonic, would no longer be adaptively resistant to antibiotics.

Interestingly, although the activity of DJK-5 in the absence of serum appeared more effective on plastic-adhered cells than those associated with the 3-D lung epithelial model, it was nevertheless able to influence both initial association with epithelial cells and biofilm growth. Furthermore, its efficacy increased substantially in combination with a low concentration of tobramycin.

In conclusion, our study demonstrated that epithelial cells influence the inhibition of bacterial association by antimicrobial agents (including commonly used antibiotics), and that this differential inhibition is in turn influenced by a common cell culture supplement, FBS. These findings highlight differences between biofilm model systems, as well as the usefulness of *in vitro* models that reflect key host characteristics. In future studies, mapping the host factors that increase or decrease the efficacy of antibiotics might provide novel therapeutic avenues aimed at modulating the host to improve treatment success.

## Experimental Procedures

### Bacterial strains, growth media and conditions

*P. aeruginosa* PAO1 (ATCC 15692) was kindly provided by Pierre Cornelis (Vrije Universiteit Brussel, Belgium) and cultured in Luria Broth (LB) overnight at 37 °C, 250 rpm for all studies. Prior to infection, overnight cultures of *P. aeruginosa* were centrifuged and resuspended in host cell culture media, i.e. GTSF-2 medium (Hyclone, Logan, UT) supplemented with 2.5 mg/L insulin transferrin sodium selenite (ITS) (Sigma-Aldrich), 1.5 g/L sodium bicarbonate (Sigma-Aldrich), and 0% or 10% heat-inactivated FBS as indicated (Invitrogen).

For live imaging of *P. aeruginosa* biofilms on the surface of host cells, an isogenic GFP-expressing *P. aeruginosa* was constructed as previously described^59^. Briefly, *P. aeruginosa* PAO1 was grown overnight at 37 °C in LB medium, washed with and resuspended in 300 mM sucrose solution at room temperature. Approximately 3 × 10^8^ CFUs of resuspended bacteria were transferred to an electroporation cuvette (2 mm gap) to which 100 ng of plasmid pUC18R6K-mini-Tn7T-Km-*gfp* and 100 ng of plasmid pTNS2 was added. After electroporation (2.5 kV, 5 ms), 1 mL LB was added where after the suspension was incubated for 1 h at 37 °C. Next, GFP-expressing *P. aeruginosa* colonies were isolated on LB agar with 50 μg/mL kanamycin. *P. aeruginosa*-GFP growth and association with 3-D lung epithelial cells was similar to the wild type strain for the duration of the experiment, except for the 17 h time point where the PAO1-GFP strain showed slightly lower association (Supplementary Figure 4).

### Antibiotics and antimicrobial peptide

The following antimicrobial agents were used in this study: the antibiotics tobramycin, gentamicin, amikacin, ceftazidine, colistin, polymyxin B and ciprofloxacin; and the cationic anti-biofilm peptide DJK-5^31^. Antibiotics were purchased from Sigma-Aldrich or TCI, while DJK-5 was manufactured to 95% purity as previously described^31^.

### Three-dimensional lung epithelial cell model

The human adenocarcinomic alveolar epithelial cell line A549 (ATCC CCL-185) was used to generate a 3-D lung model using the RWV bioreactor, as described previously^21^. GTSF-2 medium supplemented with 2.5 mg/L ITS (Sigma-Aldrich), 1.5 g/L sodium bicarbonate, and 10% heat-inactivated FBS (Invitrogen) (30 min, 56 °C) was used for A549 cell culturing. All cultures were grown at 37 °C under 5% CO_2_ conditions. Infection studies were performed using 3-D cultures grown for 11 to 14 days.

### Bacterial association with 3-D lung epithelial cells

Upon maturation of the 3-D lung epithelial cells (11–14 days), the culture medium was changed, and aggregates containing 2.5 × 10^5^ cells in 250 μL per well were transferred to 48-well plates. A culture of *P. aeruginosa* PAO1 grown for 16 hours at 37 °C in LB medium was resuspended in GTSF-2 medium and added to the 3-D host cells at an MOI of 30:1^43^. Plates were incubated statically at 37 °C under 5% CO_2_ conditions for 2 h, 6 h and 17 h. For 17 h infection studies, cultures were rinsed three times with HBSS after 1 hour of infection, where after fresh medium was supplied. This step limited the number of bacteria in the culture medium that were not associated with host cells. The rinsing step was not performed at earlier time points as bacterial association with 3-D lung epithelial cells with or without rinsing was not significantly different.

Following the respective infection times, the content of each well was transferred into new 48-well plates to avoid inclusion of bacteria attached to the plastic surface. Association of *P. aeruginosa* with host epithelial cells was assessed as described previously^43^,^51^, with modifications. Specifically, following 2 h, 6 h and 17 h infection, the 3-D aggregates were rinsed twice with HBSS, exposed to 0.1% Triton-X100, and vigorously pipetted to disrupt *P. aeruginosa* biofilms and host cells. The resulting mixture was serially diluted and plated on LB agar. Since 0.1% Triton-X100 lyses host cells and hence releases any intracellular bacteria, adhesion data at 6 h infection was compared with data obtained using 0.25% Trypsin-EDTA (which does not lyse host cells). The total CFU/mL recovered was not statistically different using either agent, thus confirming that *P. aeruginosa* did not invade polarized epithelial cells (Supplementary Figure 5). When indicated, the planktonic fraction of the culture was serially diluted and plated on LB agar for determination of CFU/mL.

### Inhibition of initial association and biofilm formation

The comparison of bacterial association of bacteria with 3-D lung epithelial cells and to plastic in the presence of antimicrobial agents was performed at the 6 h time point since we found that both host cell viability and tissue integrity (based on the formation of tight junctions) was maintained at this time point (see results section). The antimicrobial agents were diluted in cell culture medium with or without 10% FBS in 48-well plates (total volume 250 μL/well) that did or did not contain 2.5 × 10^5^ 3-D lung epithelial cells. Antibiotic concentrations were empirically determined to obtain an inhibition of adhesion to 3-D cells of at least 1 log unit (in GTSF-2 medium with 10% FBS): tobramycin 2 μg/mL, ceftazidime 8 μg/mL, colistin 2 μg/mL, ciprofloxacin 0.125 μg/mL, gentamicin 8 μg/mL, amikacin 6 μg/mL, and DJK-5 50 μg/mL. This approach allowed us to observe potential increases or decreases in antibiotic efficacy against biofilm formation on 3-D cells versus plastic. In the absence of FBS, additional concentrations of aminoglycosides were tested: tobramycin 1 μg/mL, gentamicin 4 μg/mL, and amikacin 4 μg/mL. Following addition of the antimicrobial agents, a *P. aeruginosa* culture grown for 16 hours and resuspended in cell culture medium was added to the 48-well plates (that did or did not contain 3-D lung epithelial cells) to obtain ~7.5 × 10^6^ CFU per well. Biofilms were formed in the absence or presence of the antimicrobial agents for 6 h in a 37 °C, 5% CO_2_ incubator, and the number of cells associating with 3-D lung epithelial cells was determined as described above. For determination of bacterial adhesion to plastic, bacteria were resuspended by vigorous pipetting/scraping in 100 μL of 0.1% Triton-X solution in PBS. Then, the disrupted biofilms were serially diluted and plated on LB agar.

The resulting CFU/mL values were log transformed. To determine the log inhibition of a specific treatment in the absence or presence of 3-D lung epithelial cells, the log CFU/mL of the treated biofilm was subtracted from the log CFU/mL of the no treatment control condition.

### Biofilm eradication assay on 3-D lung epithelial cells or plastic

Biofilms were grown on 3-D lung epithelial cells or on plastic as described above for 6 hours. Then, 3-D cells containing biofilms were transferred to a new well and rinsed with HBSS. Biofilms formed on plastic were also rinsed with HBSS. Next, fresh medium was supplied which contained antibiotics at the following concentrations: gentamicin at 50 μg/mL (3x MIC) and 500 μg/mL (31x MIC), amikacin at 50 μg/mL (6x MIC) and 500 μg/mL (60x MIC), and colistin at 50 μg/mL (12.5x MIC). Following addition of antibiotics, biofilms were incubated for an additional 17–18 h at 37 °C under 5% CO_2_ conditions, after which bacterial association was determined as described above.

### Light- and fluorescence microscopy

Overall morphology/integrity of 3-D lung epithelial cells, and live imaging of GFP-expressing *P. aeruginosa* biofilms was assessed with an EVOS FL Auto Imaging System (Life Technologies) or a Zeiss Axiovert.A1 Inverted Fluorescence Microscope. Imaging was performed with a 10x, 20x or 40x objective, and appropriate filter cubes.

### Immunofluorescence staining and confocal laser scanning microscopy (CLSM)

3-D lung epithelial cells infected with *P. aeruginosa* for 2 h, 6 h and 17 h were fixed with 4% paraformaldehyde (PFA) and immunostained as described previously^22^. The primary antibody used in this study targeted human β-catenin (Abcam, #ab6301, 1:100 dilution, unconjugated). A goat anti-mouse secondary antibody conjugated with AlexaFluor488 was used (Invitrogen A-11029, 1:500 dilution). Cell nuclei were stained with DAPI, incorporated in ProLong anti-fade agent (Life Technologies). Imaging was performed using a Zeiss LSM 510 Duo laser scanning microscope with a 40x objective. Image acquisition was done with the Zeiss LSM software package, and image processing with Axiovision 4.7.

### Scanning electron microscopy

Qualitative analysis of biofilm formation on the 3-D lung epithelial cell model was done with SEM. Samples were prepared as described previously^38^,^49^. Briefly, 3-D lung epithelial cells infected for 6 h were fixed with SEM fixative (3% glutaraldehyde and 0.5% PFA in PBS) for a minimum of 24 h. Then, samples were rinsed with filter-sterilized deionized water and visualized with a Philips XL 30 Environmental SEM (FEI Co., Hillsboro, Oregon). To optimize image quality, chamber pressure was adjusted between 1 and 2 Torr.

### Lactate dehydrogenase (LDH) assay and trypan blue exclusion assay

Release of cytosolic LDH by 3-D lung epithelial cells was measured using a LDH activity assay kit (Sigma-Aldrich). Medium from 3-D aggregates infected with *P. aeruginosa* for 2 h, 6 h and 17 h was centrifuged for 15 minutes at 4680 rpm to remove cell debris and bacteria. LDH release was then quantified following the manufacturer’s instructions and a standard curve using NADH was included. As positive control, 3-D lung epithelial cells containing 2.5 × 10^5^ cells were lysed with 0.1% Triton-X100. A trypan blue exclusion assay was also performed to assess host cell death, using 0.4% trypan blue solution (Sigma-Aldrich) according to manufacturer’s instructions.

### Statistical analysis

All experiments were performed at least in biological triplicate, except for SEM and CLSM imaging which was performed in biological duplicate. Statistical analysis of quantitative assays was done using SPSS statistics software version 23. The Shapiro–Wilk test was used in combination with Q/Q plot analysis to verify the normal distribution of the data. For normally distributed data, assessment of equality of variances was performed using a Levene’s test, followed by an independent sample t-test. Data sets that were not normally distributed were analyzed using a Mann–Whitney test. P-values < 0.05 were considered statistically significant.

## Additional Information

**How to cite this article:** Crabbé, A. *et al*. Antimicrobial efficacy against *Pseudomonas aeruginosa* biofilm formation in a three-dimensional lung epithelial model and the influence of fetal bovine serum. *Sci. Rep.* **7**, 43321; doi: 10.1038/srep43321 (2017).

**Publisher's note:** Springer Nature remains neutral with regard to jurisdictional claims in published maps and institutional affiliations.

## Supplementary Material

Supplementary Figures
