# Supplementary material for: Antimicrobial efficacy against Pseudomonas aeruginosa biofilm formation in a three-dimensional lung epithelial model and the influence of fetal bovine serum
Source: Sci Rep. 2017 Mar 3;7:43321. doi: 10.1038/srep43321 (PMC5335707; doi:10.1038/srep43321)
Supplement: Supplementary Figures [file srep43321-s1.pdf]

Antimicrobial efficacy against *Pseudomonas aeruginosa* biofilm formation in a three-dimensional lung epithelial model and the influence of fetal bovine serum

Aurélie Crabbé, Yulong Liu, Nele Matthijs, Petra Rigole, César De La Fuente-Nùñez, Richard Davis, Maria A. Ledesma, Shameema Sarker, Rob Van Houdt,

Robert E.W. Hancock, Tom Coenye, and Cheryl A. Nickerson

Supplementary Figure 1

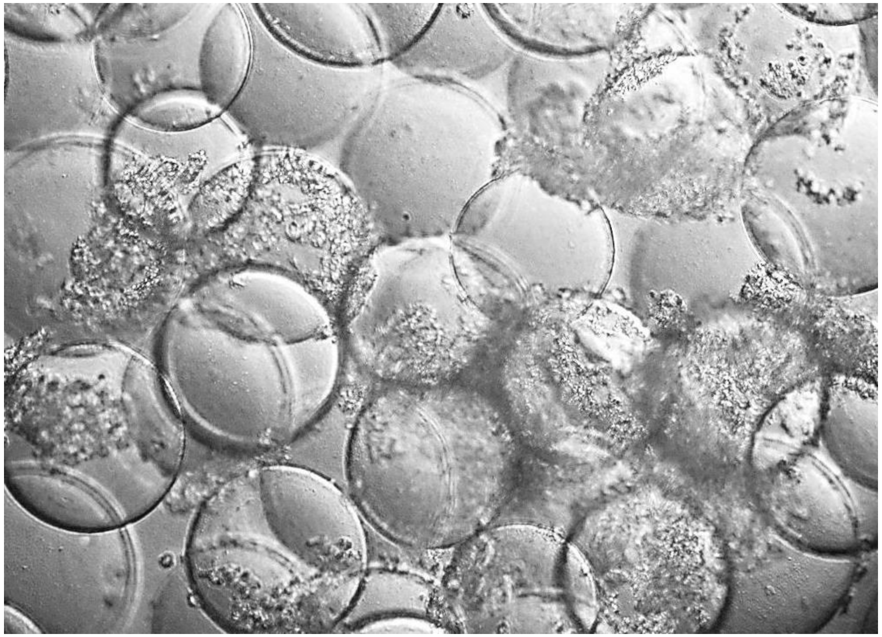

Supplementary Figure 1. Representative light microscopic image of 3-D A549 epithelial cells incubated with *P. aeruginosa* for 18 hours. Magnification = 400x

Supplementary Figure 2

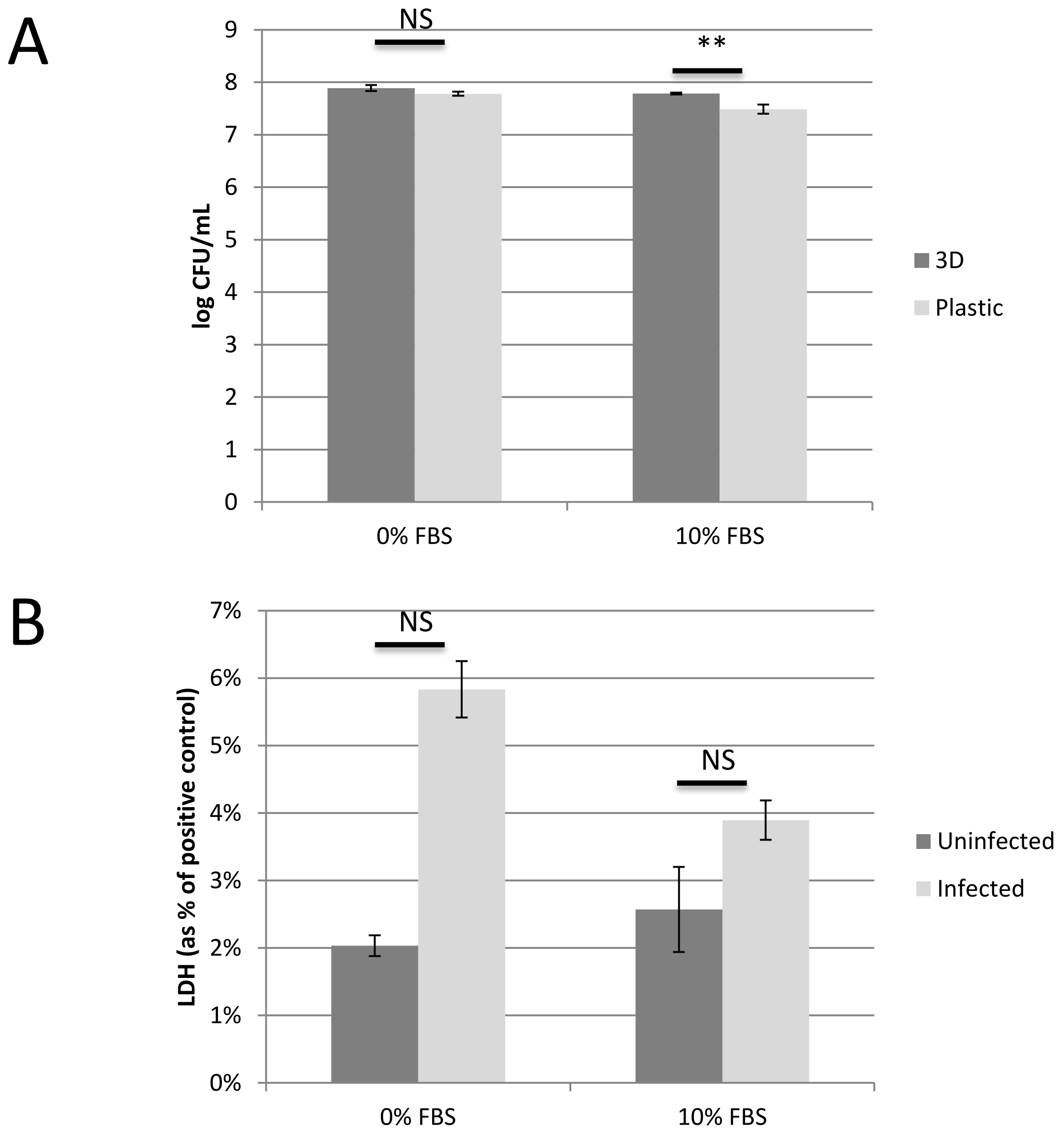

Supplementary Figure 2

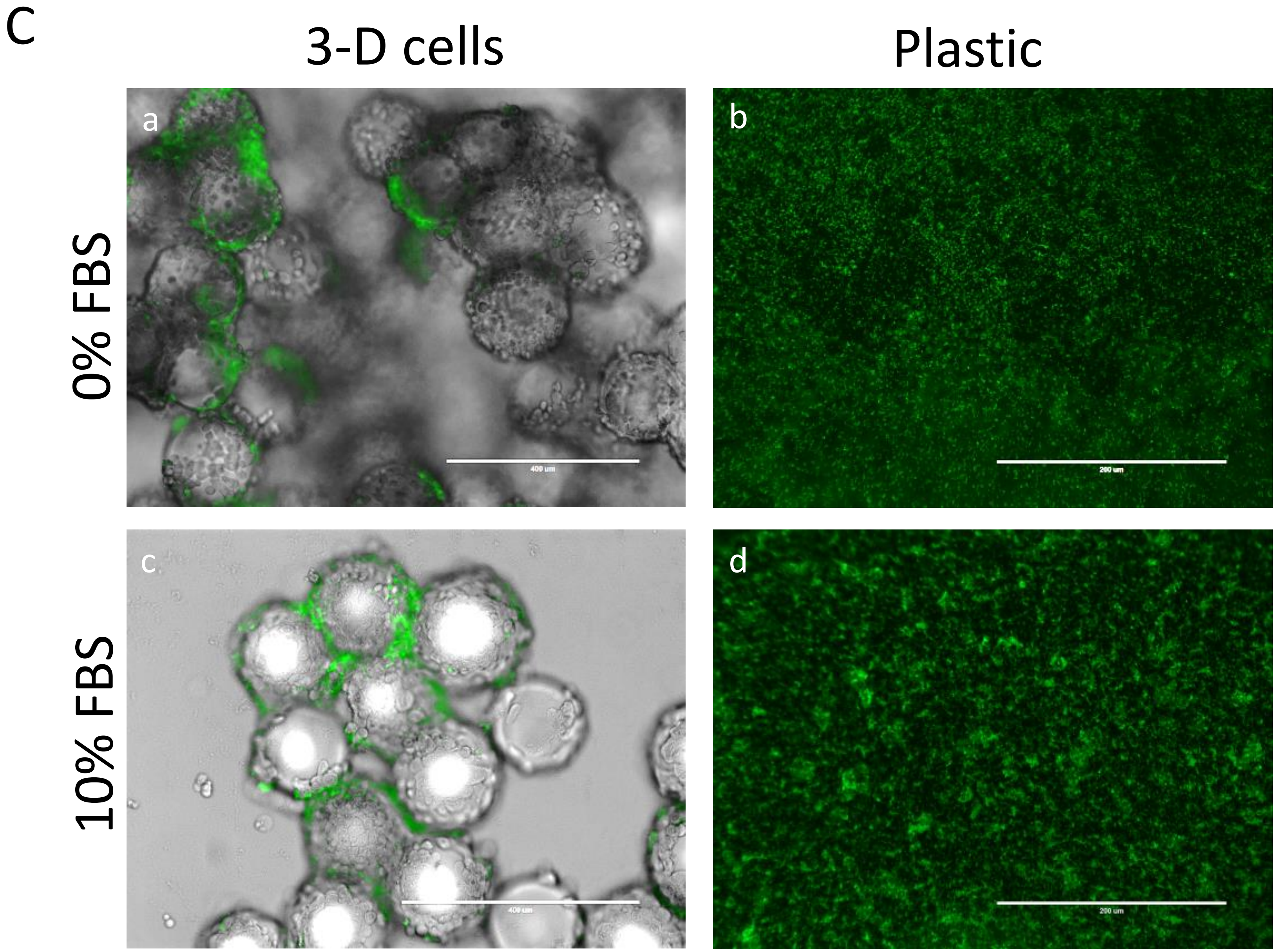

Supplementary Figure 2

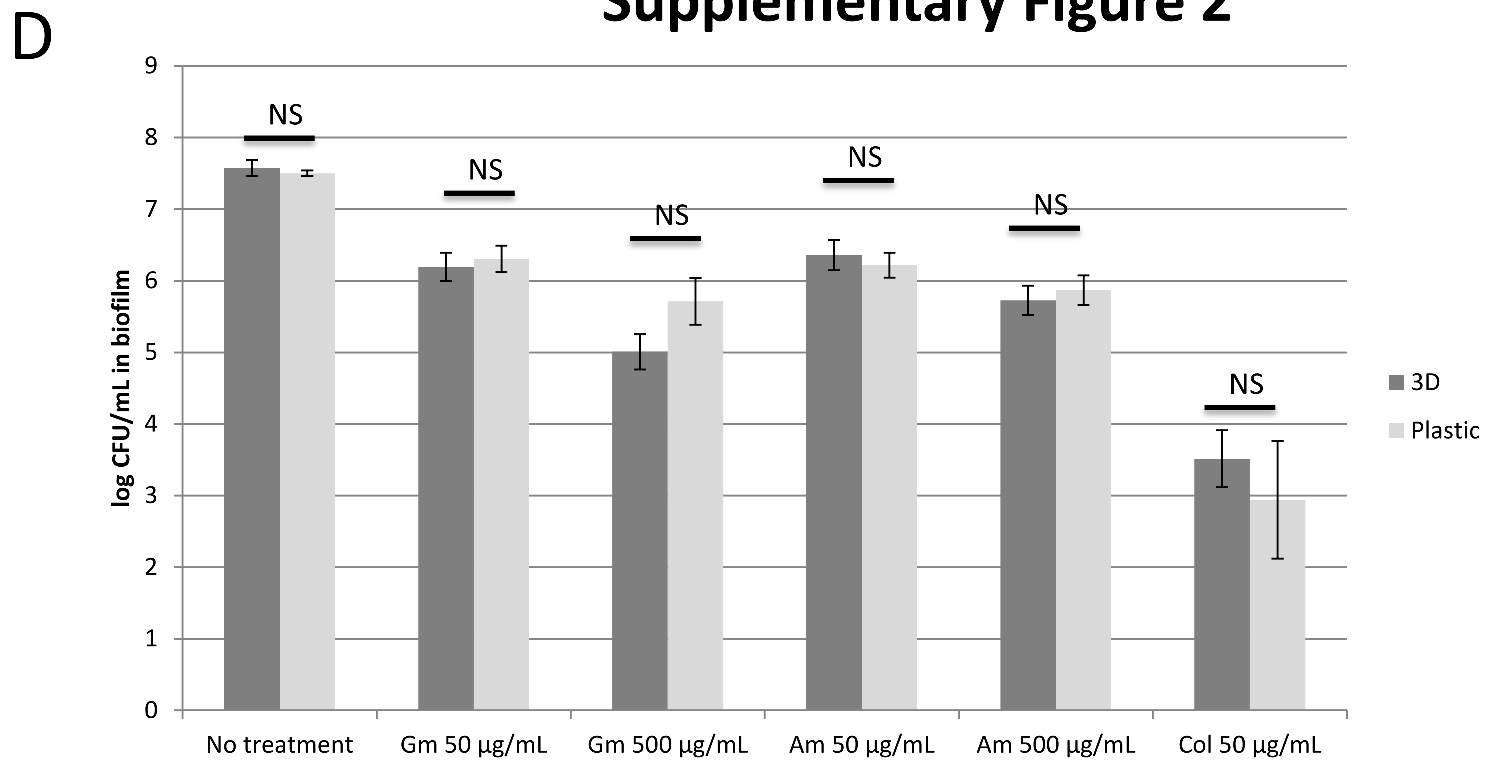

Supplementary Figure 2. Select key characteristics of biofilms grown on 3-D lung epithelial cells or plastic are not strongly influenced by FBS. (A) Bacterial association to 3-D epithelial cells or plastic after 6 h incubation. (B) LDH release of uninfected controls and cultures infected for 6 h. (C) Biofilm formation on 3-D lung epithelial cells (a and c) or plastic (b and d) by GFP-expressing *P. aeruginosa* PAO1 for 6 h. Immunofluorescence and light microscopy images at a magnification of 400x are overlaid. Magnification = 400x, (D) *P. aeruginosa* biofilms formed after 6 h on plastic or 3-D lung epithelial cells were exposed to high concentrations of antibiotics for an additional 17-18h, Control biofilms on plastic and 3-D cells were incubated for the same duration without antibiotics. Gm = Gentamicin, Col = colistin, Am = amikacin. NS = non-significant ($p > 0.05$).

Supplementary Figure 3

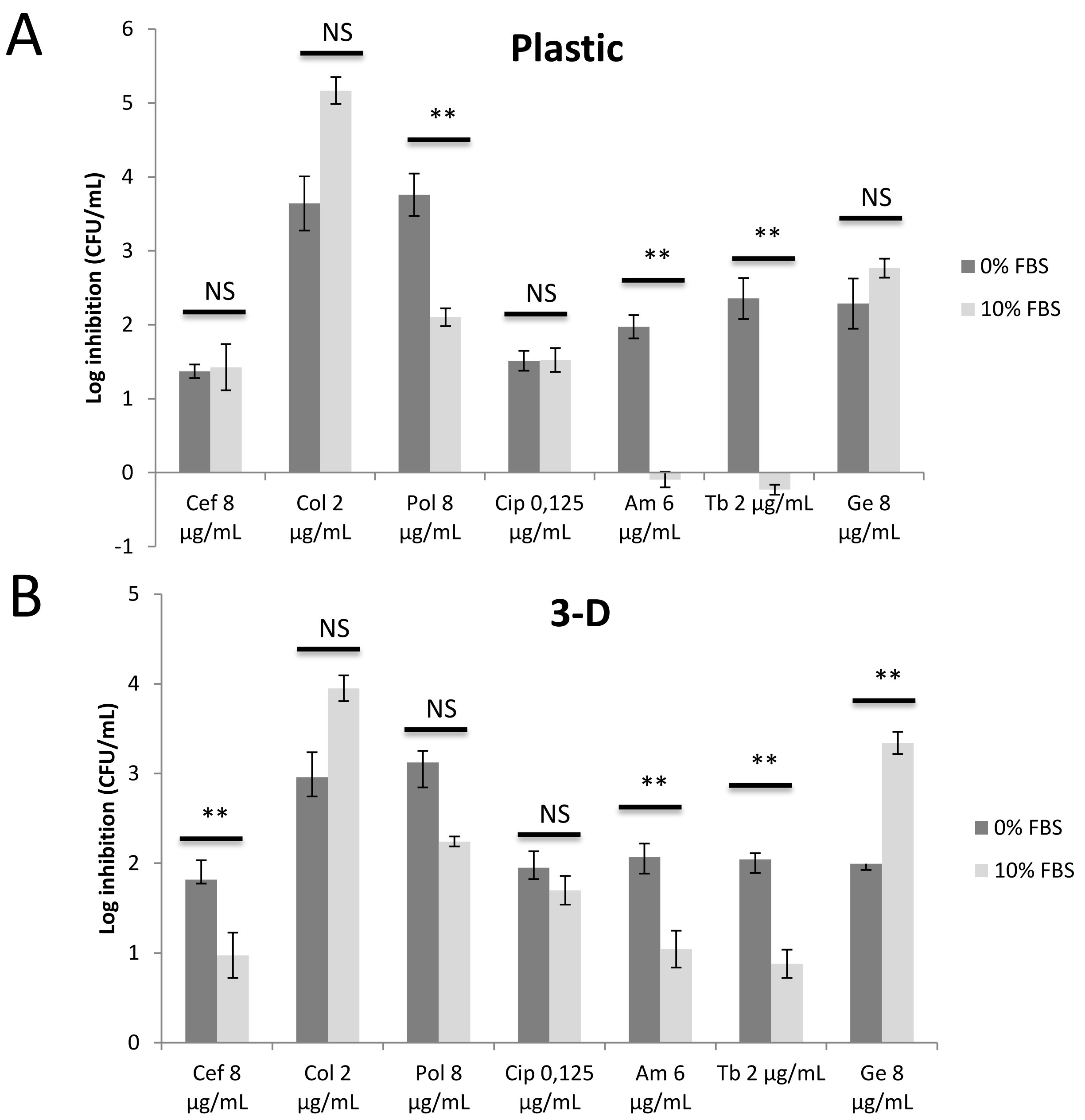

Supplementary Figure 3. Influence of 10% FBS on biofilm inhibition of antibiotics when grown on plastic (A) or 3-D lung epithelial cells (B). Data is presented as log inhibition of bacterial association compared to the control that received no treatment. Cef = ceftazidime, Col = colistin, Pol = polymyxin B, Cip = ciprofloxacin, Am = amikacin, Tb = tobramycin, Ge = Gentamicin. * $p < 0.05$, ** $p < 0.01$, NS = non-significant ($p > 0.05$). Standard error bars are presented, $n \geq 3$.

Supplementary Figure 4

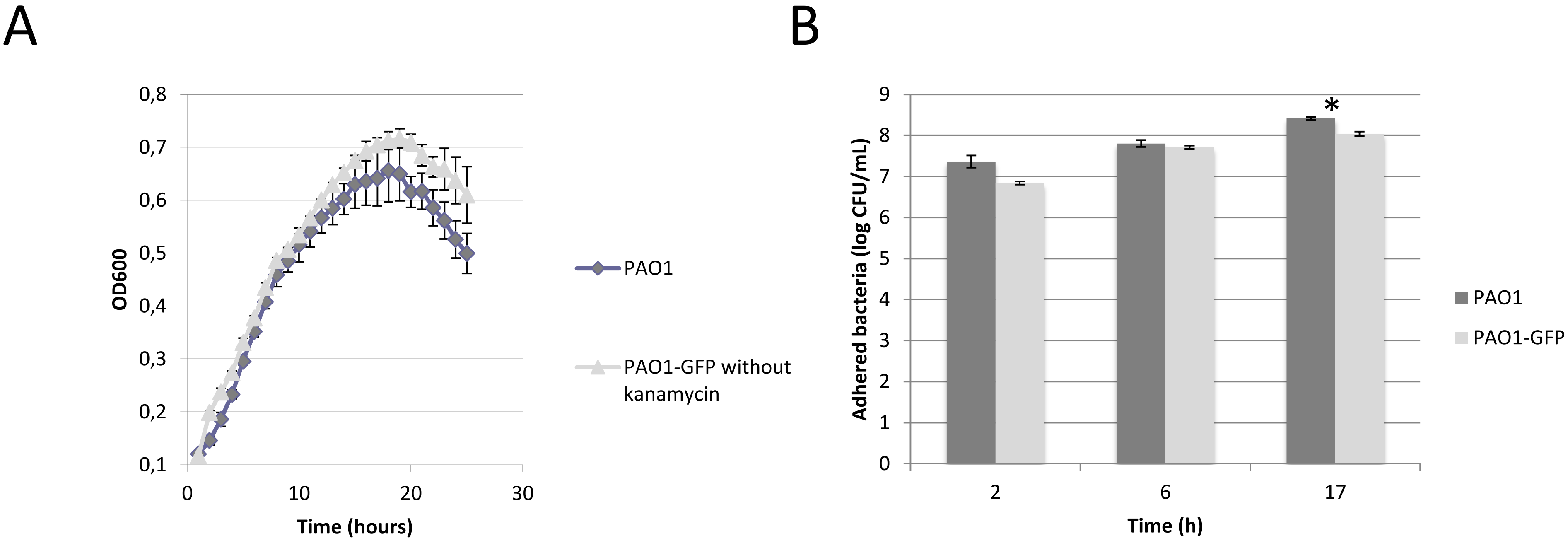

Supplementary Figure 4. Growth curves (A) and association with 3-D lung epithelial cells (B) of GFP-expressing *P. aeruginosa* PAO1 compared to wild type. Standard error bars are presented.

Supplementary Figure 5

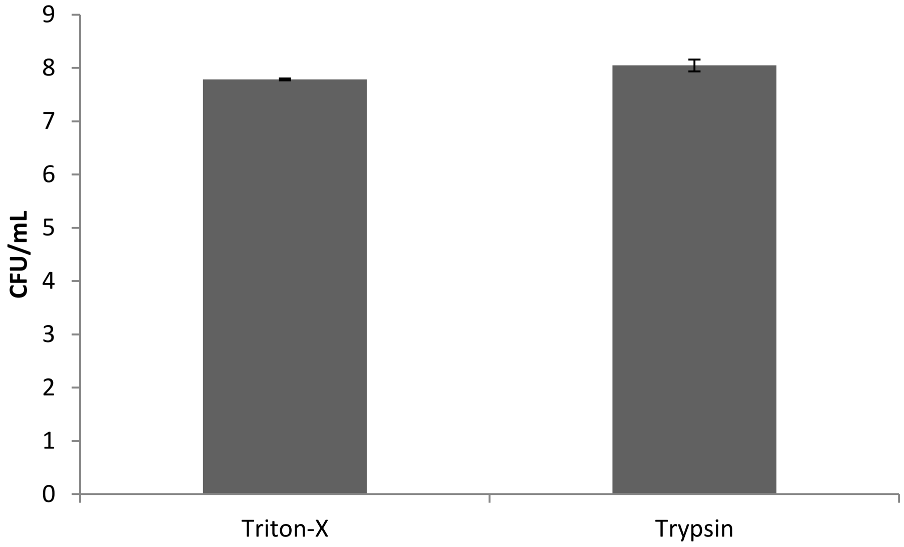

Supplementary Figure 5. Association of *P. aeruginosa* with 3-D lung epithelial cells after 6 h infection, using Triton-X100 or trypsin-EDTA.
